# Supplementary material for: MetaRibo-Seq measures translation in microbiomes
Source: Nat Commun. 2020 Jun 29;11:3268. doi: 10.1038/s41467-020-17081-z (PMC7324362; doi:10.1038/s41467-020-17081-z)
Supplement: Supplementary file 10 — Supplementary Data 7 [file 41467_2020_17081_MOESM10_ESM.zip › File2/Confidence_High_Taxonomy/98104_out.krona.html]

Javascript must be enabled to view this page.

members
magnitude
magnitudeUnassigned
count
unassigned
taxon
rank

98104\_out

2

superkingdom
2
2

phylum
1239
2

class
2
186801

order
2
186802

2
541000
family

genus
2
1263

2

SRS098571\_contig\_number\_68701SRS147022\_contig\_number\_2618
46228
species
